# Assessment of genetic diversity in Nordic timothy (*Phleum pratense* L*.*)

**DOI:** 10.1186/s41065-016-0009-x

**Published:** 2016-04-26

**Authors:** Pirjo Tanhuanpää, Maria Erkkilä, Ruslan Kalendar, Alan Howard Schulman, Outi Manninen

**Affiliations:** 1 Green Technology, Natural Resources Institute Finland (Luke), Myllytie 1, FI-31600 Jokioinen, Finland; 2 Internal Expert Services, Natural Resources Institute Finland (Luke), Humppilantie 14, FI-31600 Jokioinen, Finland; 3 Luke/BI Plant Genome Dynamics Laboratory, Institute of Biotechnology, Viikki Biocenter, University of Helsinki, P.O. Box 56, Viikinkaari 1, FI-00014 Helsinki, Finland; 4 Boreal Plant Breeding Ltd, Myllytie 10, FI-31600 Jokioinen, Finland

**Keywords:** Genetic diversity, Genetic structure, *Phleum pratense* L, REMAP, Retrotransposon marker, SSR, Microsatellite, Timothy

## Abstract

**Background:**

Timothy (*Phleum pratense* L.), a cool-season hexaploid perennial, is the most important forage grass species in Nordic countries. Earlier analyses of genetic diversity in a collection of 96 genebank accessions of timothy with SSR markers demonstrated high levels of diversity but could not resolve population structure. Therefore, we examined a subset of 51 accessions with REMAP markers, which are based on retrotransposons, and compared the diversity results with those obtained with SSR markers.

**Results:**

Using four primer combinations, 533 REMAP markers were analyzed, compared with 464 polymorphic alleles in the 13 SSR loci previously. The average marker index, which describes information obtained per experiment (per primer combination or locus) was over six times higher with REMAPs. Most of the variation found was within accessions, with somewhat less, 89 %, for REMAPs, than for SSR, with 93 %.

**Conclusions:**

SSRs revealed differences in the level of diversity slightly better than REMAPs but neither marker type could reveal any clear clustering of accessions based on countries, vegetation zones, or different cultivar types. In our study, reliable evaluation of SSR allele dosages was not possible, so each allele had to be handled as a dominant marker. SSR and REMAP, which report from different mechanisms of generating genetic diversity and from different genomic regions, together indicate a lack of population structure. Taken together, this likely reflects the outcrossing and hexaploid nature of timothy rather than failures of either marker system.

## Background

Timothy (*Phleum pratense* L.), a cool-season perennial, is the most important forage grass species in Nordic countries. Genetic diversity has been previously assessed [1] in a collection of 96 timothy accessions, of which 88 were of Nordic origin. Simple sequence repeat (SSR) markers revealed Nordic timothy accessions to be very polymorphic, having significant differences in the levels of diversity between countries, vegetation zones, and different cultivar types. However, most of the variation (94 %) existed within accessions, and no clear clustering of accessions based on any grouping was observed. This lack of resolution may either reflect the outcrossing and hexaploid nature of timothy or that SSR markers are not suitable for resolving population structure in timothy.

A wide range of DNA markers are available for diversity studies, which all have their advantages and disadvantages. SSRs are amplified from single loci, but are multiallelic and highly polymorphic. Although they are inherited codominantly, separation of different genotypes may not be possible in a polyploid species such as timothy. Therefore, each allele has to be treated as a dominant marker [1]; consequently, the markers that are amplified from the same SSR locus are not independent of each other, and consequentially information is lost. In the REMAP (retrotransposon-microsatellite amplified polymorphism) markers [2, 3] assay, the diversity is generated by the integration of retrotransposons, which move in the genome by a copy-and-paste mechanism but are fixed in position upon insertion [4]. They are ubiquitous and abundant in plant genomes, where they are dispersed on all chromosomes [5]. REMAP markers are amplified using a primer designed to a conserved retrotransposon region and another anchored to a simple sequence repeat. Products from multiple loci are produced in one PCR reaction, each with only two allele alternatives, a dominant one (amplification) and a recessive one (non-amplification). Because the mechanisms that activate retrotransposons [6] and thereby generate insertional polymorphisms are fully different than that generating SSR allelic variation (polymerase slippage) [7], the two marker systems assay different components of genetic diversity. For potato [8], alfalfa [9], and grapevine [10], retrotransposon and SSR markers in combination were shown to be highly discriminatory and effective.

In the previous study [1], a collection of 96 timothy accessions was analyzed using 13 SSRs, thus describing diversity only at this number of loci. On the other hand, these 13 SSR loci harbored as many as 499 alleles. In the present study, we used REMAP markers for studying diversity in a subset of 51 accessions and compared the results with those obtained with SSR markers. We wanted to determine if another type of marker, which would report from many more locations in the genome and assess different genomic regions where diversity is generated by a different mechanism, could describe diversity more efficiently and also reveal population structure, particularly for a polyploid species. Especially the autonomous nature of retrotransposon diversity generation and display, which is independent of the syntenic organization of polyploids, appeared suited to clonal polyploid species such as timothy. We expected that the retrotransposon markers should thereby be more likely to find genetic structure in timothy, should it exist.

## Methods

### Plant material

In the previous study [1], SSR markers were analyzed in a collection of 96 timothy accessions. Fifty-one of these were selected for the present study to be screened also with REMAP markers (Table 1). Fifteen to twenty randomly selected individuals per accession were investigated, in total 945 individuals. The number of individuals analyzed from each accession in the two studies was not exactly the same because 20 individuals had to be omitted due to their poor amplification in REMAP analysis.

**Table 1:** Fifty-one *Phleum pratense* ssp. *pratense* accessions analyzed in the study

Number code	Accession no.	Genebank	Name	Country	Cultivar type^1^	Veg. zone^2^
3	NGB10830	Nordgen	VA88119	Denmark	W	1
4	NGB10831	Nordgen	HF88266	Denmark	W	1
5	NGB15461	Nordgen	Vildbjerg AC0103	Denmark	W	1
6	NGB16650	Nordgen	Ejsing	Denmark	W	1
7	NGB1672	Nordgen	BILBO	Denmark	CV	
8	NGB1675	Nordgen	POTA	Denmark	CV	
9	NGB4053	Nordgen	SR SALTUM MH0202	Denmark	W	1
10	NGB4548	Nordgen	NR FARUP MH0202	Denmark	W	1
11	NGB132	Nordgen	LIPINLAHTI ME0901 SEP A	Finland	L	4
13	NGB14394	Nordgen	KÄRKÖLÄ HM0102	Finland	W	3
16	NGB14404	Nordgen	PAATTINEN MH0201	Finland	L	2
18	NGB14417	Nordgen	MEDVASTÖ MH0101	Finland	W	2
20	NGB747	Nordgen	NUVVUS AK0401	Finland	W	6
24	NGB1095	Nordgen	LAITASAARI ME0201	Finland	L	4
27	NGB1111	Nordgen	MÄLÄSKÄ ME0101	Finland	L	4
30	NGB1119	Nordgen	KATERMA ME0401	Finland	L	4
32	NGB2791	Nordgen	NORRGÅRD AP0101	Finland	L	3
35	NGB4066	Nordgen	TAMMISTO	Finland	CV	
36	NGB4140	Nordgen	KORPA	Iceland	L	
37	NGB4141	Nordgen	ADDA	Iceland	CV	
42	NGB7592	Nordgen	SKJØLSVIK 01-5-46-5	Norway	W	3
45	NGB10785	Nordgen	SANDBU 01-6-49-4	Norway	W	5
47	NGB17194	Nordgen	Ifjord 1-1-2-2	Norway	W	5
48	NGB17198	Nordgen	Karasjok 1-1-3-2	Norway	W	5
49	NGB2169	Nordgen	BODIN	Norway	CV	
51	NGB2180	Nordgen	GRINDSTAD	Norway	CV	
53	NGB2918	Nordgen	HUSETER 01-9-70-1	Norway	W	2
57	NGB4226	Nordgen	HATLESTAD 01-7-56-3	Norway	W	5
59	NGB4231	Nordgen	GJERDÅKER 01-7-58-1	Norway	W	5
62	NGB7548	Nordgen	NAMSVATN 01-5-40-1	Norway	W	5
64	NGB722	Nordgen	KUOSSENJARKA JP0404	Sweden	W	5
65	NGB728	Nordgen	PJESKER PH0405	Sweden	W	4
66	NGB11428	Nordgen	JONATHAN	Sweden	CV	
69	NGB14224	Nordgen	SÖNDRARP IB0101	Sweden	W	2
71	NGB731	Nordgen	RÖRMYRBERG JP0204	Sweden	W	4
73	NGB16975	Nordgen	NORRA KYLSÄTER FO0103	Sweden	W	2
76	NGB16981	Nordgen	BRÄCKETORP FO0501	Sweden	W	2
78	NGB1306	Nordgen	BRATTÅKER GB0101	Sweden	W	4
81	NGB1327	Nordgen	HAMMARN PR0401	Sweden	W	4
83	NGB1331	Nordgen	VÄSTANSJÖ SH0102	Sweden	W	4
85	NGB1537	Nordgen	ESKELHEM TL0104	Sweden	W	2
86	NGB2530	Nordgen	RÄMNE GJ0301	Sweden	W	2
87	NGB4349	Nordgen	BENESTAD JK1506	Sweden	W	1
89	PI381926	GRIN		France	P	
90	PI406317	GRIN		Russia	P	
91	IHAR151908	IHAR		Germany	P	
92	PI210426	GRIN		Greece	P	
93	PI325461	GRIN		Russia	P	
94	PI204480	GRIN		Turkey	P	
95	14G2400116	RICP		Czech Republic	P	
96	RCAT040682	RCAT		Hungary	W	

^1^ *CV* advanced cultivar, *L* traditional cultivar, landrace, *P* pending, unknown cultivar type, *W* wild population, weedy

^2^vegetation zones, according to [21]

The 51 accessions were mostly wild (30, locations in Fig. 1 in [1]); seven each were classified as landraces, cultivars, and of unknown cultivar types. Accessions were derived from all Scandinavian countries (Denmark, 8; Finland, 10; Iceland, 2; Norway, 10; Sweden, 13). In addition, eight gene bank accessions (so-called exotics) originating from non-Scandinavian countries were included in the study.

**Fig. 1:**
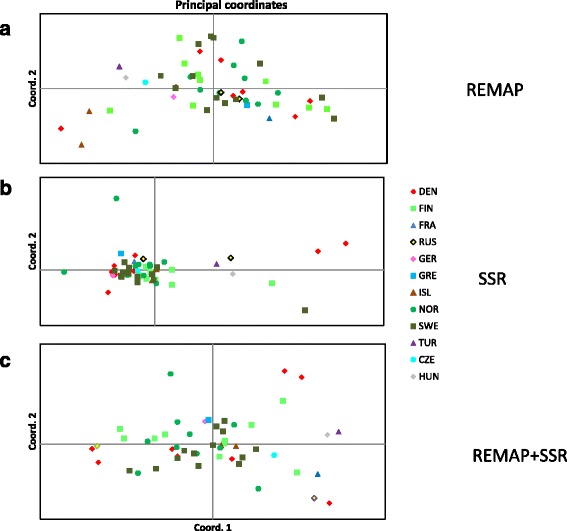
Principal coordinate analysis based on Nei’s genetic distances between accessions based on: **a** 533 REMAP markers; **b** 464 SSR markers; **c** 997 REMAP and SSR markers

### Marker analyses

DNA was extracted using the method of Tinker et al. [11] with some modifications as described in Tanhuanpää and Manninen [1]. Using the iPBS (inter- primer binding site) method, retrotransposon segments were isolated from the timothy genome, sequenced, and long terminal repeats (LTRs) identified [12]. LTR primers were designed to match conserved motifs at or near their termini, according to the methods of Kalendar et al. [13]. For REMAP marker amplification, four different retrotransposon primers (TIM1 - 4) for grasses were used. These were combined with 19 microsatellite-based primers (ISSR + number) that contain repeat units (composed of two or three bases); the 3′ ends of the primers were anchored by a single nucleotide. Because analyzing markers by gel electrophoresis is very laborious, the retrotransposon primers were labelled with a fluorescent dye, FAM (5-carboxyfluorescein), HEX (hexachloro-6-carboxyfluorescein), or TET (6-carboxytetrachlorofluorescein) to enable resolution and visualization of amplification products with a MegaBACE^TM^ 500 Sequencer (GE Healthcare, Buckinghamshire, UK).

Fifty-nine REMAP primer combinations were first tested in a small set of individuals for their functionality and efficiency to produce polymorphic bands. The four best primer combinations were chosen for final analyses (TIM1 with ISSR1, 15 and 20, and TIM2 with ISSR5). These primers, together with their sequences and properties, are shown in Table 2. The REMAP markers were amplified in a reaction volume of 10 μl, using 0.25 U of FIREPol® DNA polymerase I (Solis BioDyne OU, Tartu, Estonia), buffer B with 2.5 mM MgCl_2_ as supplied by the enzyme manufacturer, 200 μmol/L each dNTP, 10 ng of DNA, and 500 nmol/L each primer. The PCR program was run on a PTC-220 DNA Engine Dyad^TM^ Peltier Thermal Cycler (MJ Research, Waltham, MA, USA) and consisted of an initial denaturation step of 2 min at 94 °C; 32 cycles of 30 s at 94 °C, 30 s at 60 °C and 2 min at 72 °C; a final extension step of 10 min at 72 °C. After PCR, the amplified products with different labels were combined for MegaBACE runs. SSRs were developed for timothy [14], and analyses were run as described previously [1].

**Table 2:** REMAP primers that were used in the analysis of timothy diversity, their sequences and properties

Name	Sequence	nt	Tm (°C)	CG %	Linguistic complexity (%)
TIM1	GGTGCCGGCATCGATCCTTTCA	22	62.4	59.1	88
TIM2	ACGAGTGAGGACAAAGTGCGCAGA	24	61.9	54.2	79
ISSR1	ACCACCACCACCACCACCC	19	63.2	68.4	24
ISSR5	AGCAGCAGCAGCAGCAGCG	19	64.4	68.4	30
ISSR15	GTGGTGGTGGTGGTGGTGGTGA	22	64.2	63.6	28
ISSR20	TGCTGCTGCTGCTGCTGCC	19	64.6	68.4	30

*nt* nucleotides, *Tm* melting temperature, *CG %* percentage of C and G bases

### Data analyses

Each REMAP fragment represents a separate locus, and the presence and absence of the fragment was scored in a binary code (1/0). Likewise, each SSR allele was treated as a separate locus and scored in a binary code, even though SSRs are codominant markers. This was because we found the evaluation of allele dosages very unreliable in hexaploid timothy. Diversity indices for markers, including polymorphic information content (PIC), gene diversity, and major allele frequency, were calculated with the program Powermarker v3.0 [15]. A marker index (MI) for each REMAP primer combination and each SSR locus was determined by multiplying the number of polymorphic markers generated (EMF = Effective multiplex ratio) by average PIC value [16]. It illustrates the amount of information obtained per experiment (per primer combination or locus).

Genetic diversity in each accession was described with five different diversity indices: 1) the number of all markers observed (A_A_), corrected to a sample size of n = 15 with 1000 resamplings without replacement; 2) the mean number of all markers observed in each individual (A_I_); 3) the mean number of pairwise differences (PWD) (Euclidean distances) between individuals, which was counted with the program ARLEQUIN version 2.000 [17]; 4) Shannon’s diversity index *I* [18]; 5) the percentage of polymorphic loci. The last two were calculated using the program GenAlex 6.4 [19, 20]. Correlations between diversity indices based on REMAP and SSR markers, and differences in the level of diversity between different groups such as countries, vegetation zones [21], or cultivar types were determined by ANOVA Proc GLM (SAS Enterprise Guide 4.3).

The program GenAlex 6.4 [19, 20] was used to perform analysis of molecular variance (AMOVA) [22] which partitions total genetic variation to within- and among-accession variance components. The significance of the results was tested by permuting the data 999 times. Principal coordinate analyses (PCA) based on Nei’s genetic distances [23] between accessions, and a Mantel test [24], which was used to compare Nei’s distances based on REMAP or SSR data, were carried out with the software GenAlex.

## Results

### Diversity at marker loci

Four REMAP primer combinations were used for studying diversity of the 51 accessions. Because not all fragments could be read as marker peaks, selections were made on the basis of the size and shape of the peaks. The numbers of scored polymorphic markers produced by different primer combinations were as follows: TIM2 + ISSR5, 91; TIM1 + ISSR20, 84; TIM1 + ISSR1, 209; TIM1 + ISSR15, 149. A total of 533 REMAP markers were analyzed, ranging in size from 80 to 650 bp. A total of 464 polymorphic alleles in the 13 SSR loci were amplified from the 51 accessions, the number varying from 13 to 71 per accession [1]. The average diversity indices of REMAP markers were higher than those of SSR markers (Table 3) leading to a six-fold higher MI for REMAPs.

**Table 3:** Comparison of diversity measures of REMAP and SSR markers in the analysis of 51 timothy accessions

	REMAP	SSR
No. of primer combinations or loci	4	13
Total no. of markers	533	464
No. of markers per primer combination or locus = EMF^1^	133.3	35.7
PIC, average	0.131	0.086
Markers with PIC > 0.1	258 = 48 %	148 = 32 %
Markers with MAF < 0.1	365 = 68 %	371 = 80 %
Average gene diversity	0.152	0.098
Marker index (MI) = EMF x PIC	17.4	3.1

^1^effective multiplex ratio

### Genetic diversity within accessions

The observed number of REMAP markers per accession varied from 195 (PL204480) to 352 (NGB1672) (Table 4), and the number of SSR alleles from 95 (NGB10785) to 194 (NGB1111). There was only one private REMAP marker (in accession PL325461), but 43 private SSR alleles were found [1]. Diversity indices of accessions studied with REMAP or SSR markers, respectively, varied as follows: A_I_ from 47.5 (PL204480) to 84.8 (NGB1672) and from 28.4 (NGB10831) to 35.2 (NGB7592); PWD from 53.1 (PL204480) to 100.2 (NGB1672) and from 28.9 (NGB10785) to 44.9 (NGB7592); *I* from 0.159 (RCAT040682) to 0.280 (NGB1672), with mean of 0.203 ± 0.029, and from 0.109 (NGB722) to 0.156 (NGB1111), with mean of 0.138 ± 0.014; the percentage of polymorphic loci from 35.8 % (PL204480) to 64.9 % (NGB1672), with a mean of 49.0 ± 7.3 %, and from 19.8 % (NGB10785) to 41.4 % (NGB1095), with a mean of 34.4 ± 5.1 % (Table 4). The A_I_ values based on SSRs changed slightly from the previous results [1] due to exclusion of 20 individuals (see Methods).

**Table 4:** REMAP and SSR diversity in 51 timothy accessions

		REMAP (total no. of markers = 533)						SSR (total no. of markers^1^ = 464)					
Accession	No. of ind.	No. markers	A_A_^2^	A_I_^3^	PWD^4^	*I* ^*5*^	% polymorphic loci	No. markers	A_A_^2^	A_I_^3^	PWD^4^	*I* ^*5*^	% polymorphic loci
NGB10830	19	213	200.5	55.8	61.0	0.170	38.6	124	116.7	28.8	30.7	0.114	26.7
NGB10831	18	221	207.3	50.1	56.2	0.161	40.5	148	137.9	28.4	32.5	0.126	31.3
NGB15461	19	323	302.2	73.1	85.7	0.244	59.3	188	171.5	33.3	44.0	0.155	40.5
NGB16650	18	281	264.9	60.6	71.8	0.206	52.0	142	132.9	31.3	36.2	0.127	30.6
NGB1672	19	352	328.7	84.8	100.2	0.280	64.9	162	149.7	31.7	40.1	0.137	34.7
NGB1675	20	297	277.8	78.4	87.2	0.244	54.8	119	110.1	30.0	32.5	0.111	25.6
NGB4053	18	239	226.9	58.8	65.3	0.185	44.7	145	135.2	30.7	36.2	0.135	31.3
NGB4548	19	236	219.2	54.8	61.3	0.177	43.3	154	139.4	30.1	35.1	0.137	33.2
NGB132	19	313	293.0	75.6	84.7	0.242	57.2	186	167.5	31.1	39.1	0.144	39.7
NGB14394	19	276	255.6	59.3	71.2	0.204	50.8	175	162.2	32.7	42.6	0.148	37.7
NGB14404	20	295	266.0	65.7	74.5	0.214	54.6	176	158.4	33.6	40.5	0.144	37.7
NGB14417	18	213	201.4	60.7	59.4	0.166	38.8	121	115.2	28.4	32.5	0.115	25.9
NGB747	20	310	285.9	75.2	81.5	0.235	56.8	144	131.4	30.3	34.7	0.126	30.8
NGB1095	20	330	300.6	72.9	85.0	0.245	61.2	192	170.2	34.2	43.1	0.153	41.4
NGB1111	19	308	289.2	64.3	78.1	0.229	56.7	194	172.7	33.8	44.3	0.156	41.2
NGB1119	19	226	210.1	52.8	58.6	0.170	41.5	189	170.9	31.9	40.5	0.149	40.7
NGB2791	19	315	294.5	67.7	80.7	0.238	58.2	186	169.8	33.3	42.5	0.152	40.1
NGB4066	19	259	239.8	53.6	65.4	0.188	47.5	180	164.5	32.7	38.6	0.149	38.4
NGB4140	18	263	248.3	55.0	70.5	0.201	49.0	193	176.2	33.4	43.9	0.160	40.7
NGB4141	19	260	246.6	66.5	71.6	0.206	48.0	117	111.2	31.3	34.6	0.118	25.0
NGB7592	16	244	239.2	64.5	68.6	0.192	44.7	182	168.4	35.2	44.9	0.155	37.1
NGB10785	18	202	192.0	67.0	59.9	0.165	36.6	95	86.8	32.7	28.9	0.092	19.8
NGB17194	20	286	260.7	62.1	72.7	0.213	52.7	166	149.5	31.4	37.2	0.134	35.8
NGB17198	18	336	313.7	70.1	85.7	0.245	61.9	179	166.4	32.4	42.5	0.150	38.6
NGB2169	19	290	269.0	62.9	76.5	0.219	53.8	167	153.4	30.3	38.8	0.140	35.8
NGB2180	18	304	284.6	67.7	77.4	0.226	56.1	185	169.2	31.2	39.5	0.146	39.7
NGB2918	20	332	305.3	73.4	88.2	0.252	61.7	164	150.2	32.1	39.0	0.140	35.3
NGB4226	17	236	228.4	64.4	67.9	0.191	43.3	158	151.6	34.9	42.2	0.142	34.1
NGB4231	19	230	216.4	56.3	63.0	0.183	42.2	156	143.6	31.3	38.4	0.137	33.6
NGB7548	17	272	260.1	61.4	70.0	0.201	49.7	150	140.8	29.8	35.4	0.131	31.9
NGB722	19	256	241.9	77.4	71.7	0.202	45.8	115	108.8	29.5	32.7	0.109	24.4
NGB728	18	265	246.3	56.7	65.5	0.196	49.5	182	171.2	33.9	43.4	0.155	39.2
NGB11428	16	231	226.2	58.9	64.4	0.180	42.0	139	138	34.2	39.8	0.135	29.7
NGB14224	19	293	273.6	65.7	79.2	0.224	54.0	175	158.4	33.4	42.2	0.146	36.6
NGB731	20	311	288.5	79.8	84.6	0.241	57.0	182	163.2	31.9	41.1	0.150	39.2
NGB16975	19	258	237.0	56.2	66.4	0.189	47.5	176	161.8	33.5	42.2	0.150	37.7
NGB16981	15	245	245.0	59.2	69.6	0.193	44.7	153	149.8	33.6	41.7	0.138	32.1
NGB1306	18	298	280.5	64.0	76.0	0.224	55.0	184	173.6	32.9	41.4	0.152	39.7
NGB1327	19	284	267.1	70.6	79.8	0.225	52.2	162	151.4	33.6	41.2	0.144	34.9
NGB1331	19	223	208.5	57.1	60.7	0.172	40.3	170	158.4	32.8	42.1	0.144	36.4
NGB1537	16	296	290.1	68.2	82.4	0.231	54.6	139	131.7	30.5	36.1	0.125	28.9
NGB2530	19	235	218.0	57.9	61.8	0.177	42.8	165	152.8	31.9	39.6	0.141	35.6
NGB4349	20	267	244.0	62.7	70.7	0.203	49.3	171	153.4	32.4	40.6	0.141	36.9
PL381926	19	240	222.2	67.4	66.5	0.187	43.9	131	122.3	31.1	34.6	0.125	28.0
PL406317	19	243	226.8	56.5	64.1	0.183	44.5	165	151.7	31.8	38.5	0.145	35.6
IHAR151908	17	259	249.0	60.3	66.4	0.192	48.0	150	137.4	31.1	34.8	0.126	30.6
PL210426	17	245	237.0	65.5	69.4	0.194	44.8	146	138.9	31.8	38.3	0.131	30.6
PL325461	17	233	223.4	55.5	61.2	0.175	43.2	170	157.8	30.8	39.8	0.138	34.3
PL204480	19	195	182.4	47.5	53.1	0.151	35.8	158	144.3	31.6	37.6	0.133	33.6
14G2400116	19	234	220.2	57.1	65.8	0.185	42.8	186	170.5	34.2	44.0	0.153	39.9
RCAT040682	20	209	191.5	50.8	55.9	0.159	38.6	157	143.5	30.5	39.1	0.141	33.8

^1^each SSR allele treated as a separate marker

^2^corrected number of all markers in each accession

^3^mean number of all markers observed in each individual

^4^mean number of pairwise differences (Euclidean distances) between individuals in each accession

^5^Shannon’s diversity index

The strength of correlation between diversity indices based on REMAP or SSR markers varied depending on the index. No correlation existed in the level of A_I_. PWD and *I* correlated weakly at *r* = 0.27 (*P =* 0.059*)* and *r* = 0.25 (*P =* 0.073), respectively. The number of markers per accession (A_A_) correlated moderately at *r* = 0.37 (*P =* 0.0075) and the percentage of polymorphic loci strongly with *r* = 0.44 (*P* = 0.0012). Nei’s genetic distances between accessions based on REMAP and SSR data correlated strongly (*r* = 0.67, *P* < 0.001) with each other.

When studying levels of diversity between countries, vegetation zones, or different cultivar types, we found no significant differences in A_A_ and PWD based on REMAP markers (Table 5). On the other hand, statistically significant (*P* < 0.05) differences in A_A_ and PWD between different vegetation zones and in A_A_ between different cultivar types were found with SSR markers (Table 5). In the previous study with 96 accessions analyzed with SSR markers, we found significant differences (*P* < 0.05) in levels of diversity in all groups [1]. When the total number of markers was studied on an individual rather than accession level (A_I_), significant differences for each grouping and with both marker types were discovered (Table 5). However, these differences explained only a minor fraction of variation between individuals (1 to 5 %).

**Table 5:** ANOVA tables indicating *F*-values, significance levels *P,* and R^2^ for comparisons between different groups for their levels of REMAP and SSR diversity

REMAP		Total no. of markers (A_A)_			No. of pairwise differences (PWD)			Number of markers per individual		
Diversity index	df	F	*P*	R^2^	F	*P*	R^2^	F	*P*	R^2^
Grouping										
Accession	50							6.11	<0.001	0.25
Country	5	1.73	0.147	0.16	1.49	0.210	0.14	5.08	<0.001	0.03
Vegetation zone	5	0.74	0.602	0.11	0.74	0.600	0.11	5.08	<0.001	0.04
Cultivar type	2	2.03	0.144	0.09	1.96	0.153	0.09	3.98	0.019	0.01
SSR		Total no. of markers (A_A)_			No. of pairwise differences (PWD)			Number of markers per individual		
Diversity index	df	F	*P*	R^2^	F	*P*	R^2^	F	*P*	R^2^
Grouping										
Accession	50							3.18	<0.001	0.15
Country	5	1.15	0.348	0.11	1.53	0.200	0.15	5.28	<0.001	0.03
Vegetation zone	5	3.90	0.008	0.40	3.49	0.014	0.38	6.67	<0.001	0.05
Cultivar type	2	4.70	0.015	0.19	1.71	0.194	0.08	4.98	0.007	0.01

### Genetic divergence between accessions and groups

AMOVA was performed in order to divide the total genetic variation into three components: variation within accessions, among accessions, and among countries. Most of the variation in the studied material was found within accessions: 89 % when based on REMAP markers, 93 % when based on SSR markers, and 91 % when based on both marker types (Table 6).

**Table 6:** Analysis of molecular variance in 51 timothy accessions based on 533 REMAP markers, 464 SSRs or both

		REMAP				SSR				REMAP and SSR			
Source	df	SS	MS	Variance components	% total	SS	MS	Variance components	% total	SS	MS	Variance components	% total
Among countries	5	896.84	179.37	0.42	1	574.09	114.82	0.21	1	1471.68	294.34	0.63	1
Among accessions/countries	45	5181.97	115.15	4.21	10	3719.53	82.66	2.45	6	8901.50	197.81	6.66	8
Within accessions	894	33312.58	37.26	37.26	89	33271.32	37.22	37.22	93	66583.90	74.48	74.48	91
Total	944	39391.39		41.89	100	37564.94		39.88	100	76957.08		81.77	100
Stat	Value					Value				Value			
PhiRT	0.010					0.005				0.008			
PhiPR	0.101					0.062				0.082			
PhiPT	0.110					0.067				0.089			

Probability, P(rand ≥ data), for PhiRT, PhiPR and PhiPT = 0.001, and is based on permutation across the full data set

PhiRT = AC / (WA + AA + AC) = AC / TOT

PhiPR = AA / (WA + AA)

PhiPT = (AA + AC) / (WA + AA + AC) = (AA + AC) / TOT

Key: AC = est. var. among countries, AA = est. var. among accessions, WA = est. var. within accessions

No genetic divergence was observed between vegetation zones or cultivar types either using SSR or REMAP markers or both (AMOVA, *P* < 0.05), which might be due to the small numbers of members in different classes. However, the same result was obtained with SSR markers when 96 accessions were studied [1]. In PCA analysis as well, no clustering of accessions based on countries, vegetation zones, or cultivar types was seen (Fig. 1). The first two axes respectively explained 44.1 %, 45.8 %, or 41.1 % of the variation when REMAPs, SSRs, or both marker types were used in the analysis.

## Discussion

Previously, SSR markers revealed timothy to be very diverse both on the individual and accession level when studied in a collection of 96 accessions. Because it was impossible with SSRs to resolve any population or geographical structure [1], we here have applied a very different kind of neutral marker, REMAPs, which are based on displaying retrotransposon insertions.

Both REMAPs and SSRs were highly polymorphic. Variation was observed mostly within accessions but with slightly smaller proportion for REMAPs (89 % vs. 93 %). This difference may be due to the biology of how SSR and retrotransposon polymorphisms are generated. SSRs are generated by replication slippage [7], a process expected to be independent of the environment. In contrast, retrotransposons are known to be activated by both biotic and abiotic stresses [6], conditions which may well be greater in some populations compared with others. Population-level stress would thereby lower the proportion of polymorphism on the individual level and increase it on population or geographic levels.

Diversity indices in accessions were lower for SSR than for REMAP markers. This is likely because SSR markers (i.e., alleles) are not independent of each other; there is a theoretical maximum number of markers that can exist in one individual. If all SSR loci would amplify from all three genomes of *Phleum,* the maximum number of markers would be 78 (13 loci, 6 alleles in each). However, there is evidence that timothy is an allopolyploid [25]. Allopolyploidy is consistent with our earlier results [1], with some SSR loci found only in one genome whereas others were present in all three. Therefore, the real maximum number of SSR alleles in any one individual lies somewhere between 26 and 78. In the present study, the observed maximum was 45.

Polyploids represent about 50 % of flowering plants [26]. In polyploids, the problem of lack of independence between SSR loci is particularly a problem, but given a very high number of loci developed from the genome sequences of major crops such as cotton or wheat, chromosome-specific markers can be recovered [27]. For agricultural species without reference genomes such as timothy or for many wild species [28], selection of markers with diploid inheritance can reduce the usable loci to very low numbers.

In contrast to SSRs, no limit exists for the maximum amount of REMAPs in one individual because retrotransposon insertions are independent of each other. Moreover, different retrotransposon families, such as in the hexaploid wheat genomes [29], show different evolutionary histories, enabling discrimination between homeologues. Retrotransposon markers have been deployed effectively for even the highly polyploid sugarcane [30]. Although codominant REMAPs also exist, codominance does not restrict the possibility of co-existence of markers in one individual. The maximum amount for REMAPs observed in one individual in the present study was 121. Correlations between diversity indices based on REMAP or SSR markers were mostly low or moderate because the two marker systems report from different genomic regions where polymorphisms are generated by different processes. On the other hand, even though SSRs could be treated as codominant markers, it has been suggested that large similarities between diversity indices with dominant markers but somewhat lower between dominant markers and SSRs are due to insufficient numbers of analyzed SSR loci [31].

When using markers for measuring distances, PWD between individuals correlated weakly (*r* = 0.26) but genetic distances between accessions strongly (*r* = 0.67) between the two marker types. PWD is based on the Euclidean distances between individuals whereas distances between accessions are based on marker frequencies. The same sort of result – poor or nonexistent individual-by-individual correlations but moderate correlation between accessions – was obtained when amplified fragment length polymorphisms (AFLPs), which are comparable to REMAPs by being a multilocus and dominant marker type, and SSRs were compared [32]. In potato, a low correlation of SSR and REMAP markers (*r* = 0.17) in the Mantel’s matrix correspondence test was found [8].

Comparing the two marker types, REMAP markers were more cost-efficient. The PCRs of four REMAP primer combinations were made separately, and products from two different combinations with different fluorescent labels were combined for MegaBACE runs. As a consequence, for the whole diversity analysis study (945 samples), 40 PCRs on 96-well microtitre plates were made and analyzed in 20 Megabace runs. A total of 533 polymorphic markers was produced. On the other hand, the 13 SSR loci were multiplexed into 5 PCR reactions and analyzed in 5 MegaBACE runs, requiring in total 50 PCR plates and 50 MegaBACE runs. In addition, some planning and optimization was required in order to multiplex the PCR reactions for the various SSR loci. A total of 464 SSR markers (i.e., alleles) was amplified. Accordingly, more REMAP markers (i.e., loci) were produced with less labor, money, and time. The MI was over six-fold higher with REMAPs, which is not due only to the need to interpret SSR alleles as separate markers but is also typical for markers with an effective multiplex ratio, and has been detected also when AFLPs have been compared with SSRs [16].

Knowledge of genetic variation and relationships between individuals and accessions is essential when conserving and using genetic resources. Evaluation of genetic diversity requires analysis of multiple markers as efficiently as possible. Choosing a suitable marker type, several aspects have to be taken into account, not only expected heterozygosity and marker index, but also technical difficulty, ease of genotyping, cost, and availability. Technically, there were no differences between REMAPs and SSRs and we encountered analysis difficulties with both marker types. All SSR peaks contained some degree of stutter, which complicated the identification of alleles. On the other hand, interpretation of REMAP markers was very slow because several markers were amplified in one PCR reaction and there was a wide variation in peak heights. All peaks could not be analyzed and selections had to be made according to peak heights and frequency. Difficulties in scoring hindered the use of automated analysis programs for both marker types. Regarding availability, there are universal retrotransposon primers that can be used in any species, and primers specific for Graminae also have a vast range of use. Moreover, SSRs have not been developed for every species, and transform rates from one species to another depends on the genetic distance of the taxa [33]. These general conclusions regarding the utility of SSR and retrotransposon markers alone and in combination are consistent with those for four diverse dicot species, distant from the monocot timothy [8–10].

## Conclusions

When diversity in a polyploid species is examined, where the codominant nature of SSRs is of no use, dominant REMAP markers, as analyzed by size on a sequencer, were more cost-efficient. REMAPs also described diversity from a larger segment of the genome compared to the same number of SSR alleles. On the other hand, SSRs detected differences in the level of diversity in different groups better than REMAPs. Furthermore, private SSR alleles were found, making SSRs better for accession identification. Private alleles, however, can be developed from retrotransposon markers using the RBIP (retrotransposon-based insertion polymorphism) and ISBP (insertion site-based polymorphism) methods, which are locus-specific [34]. Genetic distances between accessions were similar with REMAP or SSR markers, but neither marker type could reveal any clear divergence between vegetation zones, cultivar types or countries in the polyploid, very polymorphic and heterozygous timothy species. SSR and REMAP polymorphisms derive from very different mechanisms. Variations in SSR numbers at individual loci derive from polymerase slippage during replication. In contrast, retrotransposon insertions, which can be stress-driven, generate the priming sites for retrotransposon-based marker methods. Given the vastly different numbers of microsatellite and retrotransposon loci queried by the marker systems used, which report from very different genomic regions, the fact that they together show a lack of structure likely reflects the outcrossing and hexaploid nature of timothy rather than failures of either marker system. Both retrotransposons and SSRs, however, are neutral markers; patterns of variation in the gene space of timothy, such as through single nucleotide polymorphism (SNP) genotyping, remain to be explored. These would allow the possibility to evaluate allele dosages, thereby increasing the information embodied in each locus. SNP markers have been used in sugarcane, a complex autopolyploid species, to estimate ploidy level and also the dosage of SNPs [35]. The availability of SNP markers has increased with the invention of the genotyping by sequencing strategy (GBS) [36] and a recent study presents its use to evaluate allele frequencies in populations in an outbreeding species, perennial ryegrass [37]. Such techniques could be applied to timothy as well to study the structure of accessions. On the other hand, the importance of using both molecular and phenotypic markers for assessing diversity especially when evaluating adaptive potential has been emphasized in a study where timothy accessions were characterized with SSRs, chloroplast DNA sequences, as well as by morphological and phenological traits [38].
